# Global prevalence and distribution of coinfection of malaria, dengue and chikungunya: a systematic review

**DOI:** 10.1186/s12889-018-5626-z

**Published:** 2018-06-08

**Authors:** Nasir Salam, Shoeb Mustafa, Abdul Hafiz, Anis Ahmad Chaudhary, Farah Deeba, Shama Parveen

**Affiliations:** 1 College of Medicine, Al-Imam Mohammad Ibn Saud Islamic University (IMSIU), Riyadh, Saudi Arabia; 2 Department of Parasitology, College of Medicine, Umm Al-Qura University, Mecca, Saudi Arabia; 3 Centre for Interdisciplinary Research in Basic Sciences, Jamia Millia Islamia, New Delhi, 110025 India

## Abstract

**Background:**

Malaria, Dengue and Chikungunya are vector borne diseases with shared endemic profiles and symptoms. Coinfections with any of these diseases could have fatal outcomes if left undiagnosed. Understanding the prevalence and distribution of coinfections is necessary to improve diagnosis and designing therapeutic interventions.

**Methods:**

We have carried out a systematic search of the published literature based on PRISMA guidelines to identify cases of Malaria, Dengue and Chikungunya coinfections. We systematically reviewed the literature to identify eligible studies and extracted data regarding cases of coinfection from cross sectional studies, case reports, retrospective studies, prospective observational studies and surveillance reports.

**Results:**

Care full screening resulted in 104 publications that met the eligibility criteria and reported Malaria/Dengue, Dengue/Chikungunya, Malaria/Chikungunya and Malaria/Dengue/Chikungunya coinfections. These coinfections were spread over six geographical locations and 42 different countries and are reported more frequently in the last 15 years possibly due to expanding epidemiology of Dengue and Chikungunya. Few of these reports have also analysed distinguishing features of coinfections. Malaria/Dengue coinfections were the most common coinfection followed by Dengue/Chikungunya, Malaria/Chikungunya and Malaria/Dengue/Chikungunya coinfections. *P. falciparum* and *P. vivax* were the commonest species found in cases of malaria coinfections and Dengue serotype-4 commonest serotype in cases of dengue coinfections. Most studies were reported from India. Nigeria and India were the only two countries from where all possible combinations of coinfections were reported.

**Conclusion:**

We have comprehensively reviewed the literature associated with cases of coinfections of three important vector borne diseases to present a clear picture of their prevalence and distribution across the globe. The frequency of coinfections presented in the study suggests proper diagnosis, surveillance and management of cases of coinfection to avoid poor prognosis of the underlying etiology.

**Electronic supplementary material:**

The online version of this article (10.1186/s12889-018-5626-z) contains supplementary material, which is available to authorized users.

## Background

In recent years the spread of vector borne diseases has gained concern worldwide, especially in tropical and subtropical regions because of their recurring outbreaks [1]. Some of these diseases have become endemic in many areas causing millions of cases every year [2]. The most common of these diseases includes Malaria, Dengue and Chikungunya spread by mosquito bites. Malaria has been long recognized as a significant public health threat with around 212 million cases reported in 2015 alone [3]. Malaria is caused by five different species of Protozoal parasite, *Plasmodium*. These include *P. falciparum, P. ovale, P. malariae, P. vivax and P. knowlesi* that are carried and spread by *Anopheles* mosquito [4, 5]. Dengue and Chikungunya are caused by viruses named Dengue virus (DENV) and Chikungunya virus (CHIKV) respectively. Both are spread by common mosquito vectors *Aedes s p.* Dengue viruses have four serotypes DENV-1, 2,3 and 4 [6]. As many as 400 million people are affected with Dengue every year [7]. Chikungunya follows somewhat unique pattern of spread across the world, it has the potential to emerge and re-emerge, drastically affecting a population and then remaining undetected for years [8]. In recent years many tropical countries have seen an unexpected rise and spread in cases of Dengue and Chikungunya [9].

These three vector borne diseases share an overlapping epidemic pattern with most cases reported from tropical regions of the world. Several studies have been published reporting co-circulation of Malaria, Dengue and Chikungunya [10, 11]. Apart from shared endemicity, the three diseases also share similar clinical presentation with febrility as the most common symptom. There are several distinguishing features also, like periodic increase and decrease of fever in Malaria, hemorrhagic conditions and depletion of platelet count in Dengue and severe arthralgia in case of Chikungunya infection [12, 13]. The cumulative burden of these infections has increased in recent times with frequent outbreak of Dengue and Chikungunya being reported from several parts of the world. Global travel and rapid urbanisation are important factors that have contributed in expansion of disease endemicity by introducing the vector population to exotic surroundings [14].

Simultaneous infections with more than one infectious agent complicate the diagnosis and course of treatment available. Due to the similar nature of initial symptoms for Malaria, Dengue and Chikungunya and overlapping endemicity, misdiagnosis of dual infection as monoinfection is a real possibility. Indeed several reports have been published reporting such scenarios. These arthropod borne diseases affect some of the poorest countries and in resource poor settings; clinician might rely on symptoms and endemicity for diagnosis, which might lead to underdiagnosis of cocirculating pathogens [15]. Despite similar clinical presentation the course of treatment is entirely different for all three diseases. Malaria is treated using antimalarial drugs. In case of Dengue and Chikungunya no vaccine or drug is available and clinicians rely on supportive therapy [13, 16]. Any delay in either diagnosis or start of therapy for any of these infections could have fatal outcomes. Also, there is lack of sufficient information on how concurrent infections affect disease severity and outcome. Several studies have been published that report cases of concurrent infection with two of these pathogens and in rare instances concurrent infection with all three vector borne infections. Such reports have the potential to inform public health officials and clinicians about the prevalence, disease severity and treatment options available for concurrent infections. The purpose of the present review is to assess the prevalence of such infections by thorough search and analysis of published literature.

## Methodology

### Search strategy

We did a review based on PRISMA (Preferred Reporting Items for Systematic Reviews and Meta-Analyses) guidelines to identify all relevant publications pertaining to the prevalence of Malaria, Dengue and Chikungunya coinfection. We systematically searched PubMed and Web of Knowledge from inception up to April 2018, using the following search terms anywhere in the articles: Malaria AND Dengue or Malaria AND Chikungunya or Dengue AND Chikungunya. We searched without any bar on language, publication or nature of studies. To identify additional studies, reference list of publications were carefully screened.

#### Eligibility criteria

Initial assessment was based on review of title and abstract of all studies. Full texts of potentially relevant studies were further analysed for coinfection prevalence data. Cross-sectional studies, retrospective analysis and case reports with full text availability and reporting data about any/all of the coinfections were included in the study. We excluded studies carried out in animals, reviews, letters, opinion pieces, grey literature, dissertations and conference abstracts.

#### Data extraction

The data extracted from the selected publications included first author, date of survey, place where the study was carried out, sample size and age, type of diagnostic testing performed, study design and prevalence of coinfection. All the data was entered in an excel file and double-checked.

### Prevalence mapping

The extracted data was used to create a map of prevalence of coinfection cases. All the cases reported were from seven geographical locations, South Asia, Africa, Southeast Asia, South America, North America, Caribbean and the Middle East. A total of 19 countries reported cases of Malaria/Dengue coinfection; while 24 countries reported coinfection cases of Dengue/Chikungunya. Malaria/Chikungunya cases were reported from 6 countries. Malaria/Dengue/Chikungunya coinfections were reported from only 3 countries. The maps were created using openly available maps (https://www.freeworldmaps.net).

## Results

**Fig. 1:**
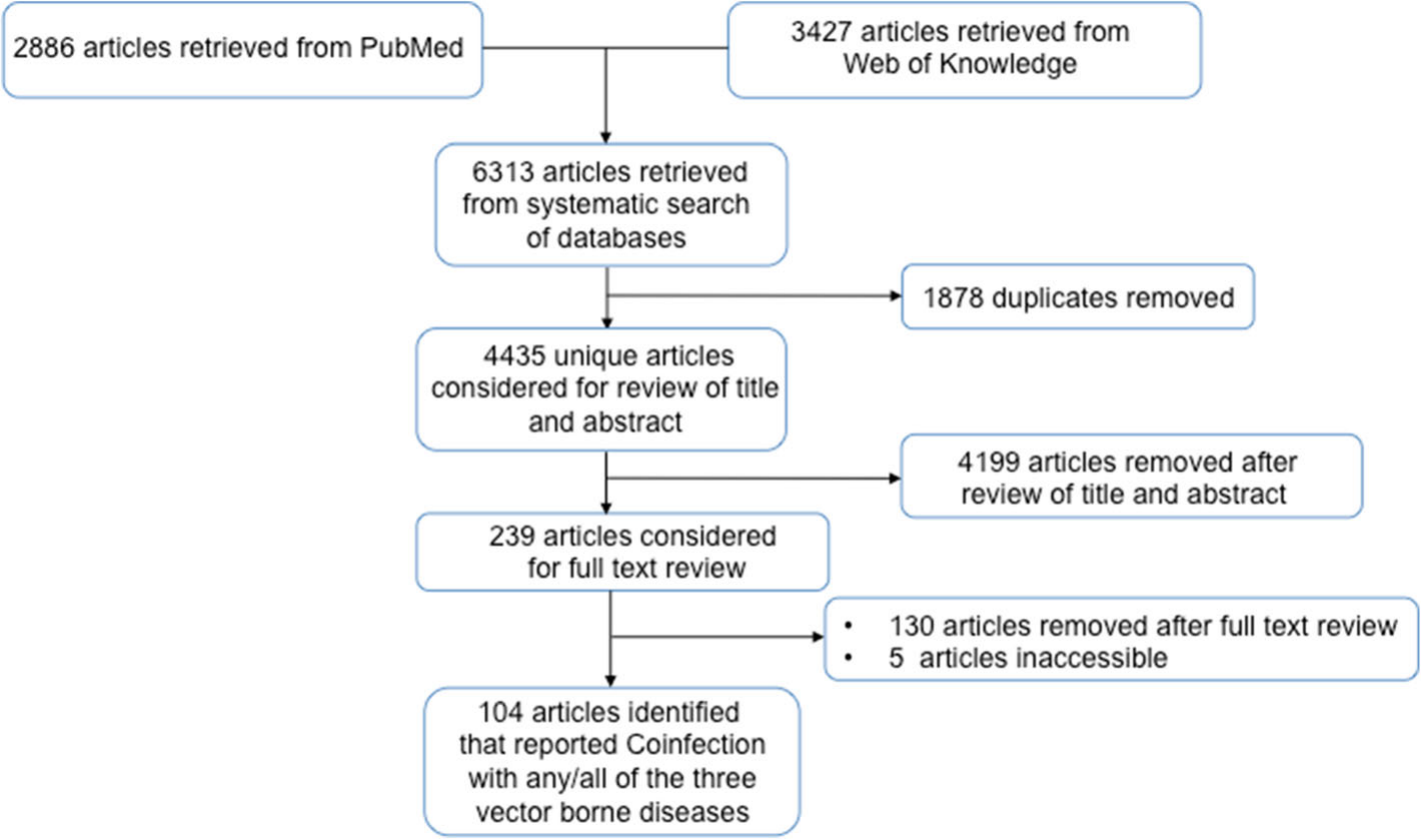
Schematic representation of the study selection process

We were able to identify 109 publications that reported the data for any coinfections (Fig. 1, Additional file 1: Table S1). The full text of 104 publications were available out of which 48 were cross sectional studies, 37 were case reports, 13 were retrospective analysis, 5 were prospective studies and 1 surveillance report [17–120]. 49 studies reported only Malaria/Dengue coinfections (Table 1) while 44 studies reported only Dengue/Chikungunya coinfections (Table 2). 1 study reported only Malaria/Chikungunya infection. 3 studies reported both Malaria/Dengue and Malaria/Chikungunya coinfections (Table 3) and 1 study reported Malaria/Dengue, Dengue/Chikungunya and Malaria/Chikungunya coinfections. Malaria/Dengue/Chikungunya coinfections were reported by 4 separate studies (Table 4). 2 studies reported Malaria/Dengue, Dengue/Chikungunya, Malaria/Chikungunya and Malaria/Dengue/Chikungunya coinfections. All of the studies, except two, were published after year 2005. Cases of coinfections were reported from all age groups and two studies from India and Burma reported data from only pregnant females. Blood smear was the most prevalent method for detection of Malaria parasite, while NS1 (Non-structural protein-1) and immunoglobulin ELISA were the most common methods for the detection of Dengue. IgM ELISA was the predominant method for the detection of most cases of Chikungunya. In 14 studies *P. falciparum* was the cause of Malaria while another 13 reported *P. vivax* as the infecting species alongside coinfecting arbovirus. 12 studies reported both *P. falciparum* and *P. vivax* with Dengue virus in the same population. Another 5 studies reported *P. falciparum*, *P. vivax* and Dengue virus in the same individuals. *P. knowlesi* was reported by two studies and *P. ovale* was reported by one study.

**Table 1:** Coinfection cases of Malaria and Dengue

S.No.	Citation	Place	Year	Study design	N	Positive for coinfection	Coinfection (%)	Age	Diagnostic test ML/DN	Remarks
South Asia										
1	Abbasi [17]	Karachi, Pakistan	Sept.2007-Jan. 2008	Cross sectional	112	26	23	13–70	Blood smear / IgM and IgG ELISA	*P. vivax- 25, P. falciparum*- 1
2	Ahmad [18]	Uttarakhand, India	Dec 2012-Dec2013	Retrospective observational studies	233	9	3.8	38.6 ± 16	Blood smear/ IgM ELISA	NM
3	Alam [19]	Patna, India	2013	Case report	1	1	NA	42	Blood smear /NS1, IgM and IgG ELISA	*P. falciparum*
4	Ali [20]	Rawalpindi, Pakistan	Nov. 2003-Oct. 2004	Cross sectional	800	9	1	17–50 years	Blood smear /IgM ELISA	*P. vivax-8, P. falciparum-1*
5	Arya [21]	Delhi, India	2003	Case report	2	2	NA	35 and 63 years	Blood smear /IgM ELISA	*P. vivax*
6	Assir [22]	Lahore, Pakistan	Aug- Nov 2012	Cross sectional	856	17	2	12–32	Blood smear /PCR, NS1 and IgM ELISA	*P. vivax* - 14, *P. falciparum*-3
7	Barua [23]	Mumbai, India	June-Nov. 2014, June -Nov. 2015	Retrospective analysis	573	44	8	NM	Blood smear / NS1 and IgM ELISA	NM
8	Bhagat [24]	Mumbai, India	2014	Case report	3	3	NA	8 months −12 year	Blood smear, RDT/NS1, IgM and IgG ELISA	*P. vivax*
9	Bhalla [25]	Delhi, India	2006	Case report	1	1	NA	21	Blood smear /IgM ELISA	*P. falciparum*
10	Chander [26]	Chandigarh, India	2009	Case report	1	1	NA	28	Blood smear /IgM ELISA	*P. falciparum*
11	Deresinski [27]	USA, infected in India	2003, Dec	Case report	1	1	NA	27	Blood smear/IgM and IgG ELISA	*P. vivax*
12	Faruque [28]	Chittagong, Bangladesh	Dec. 2008-Nov. 2009	Cross sectional	720	1	0.1	All ages	RDT/IgM ELISA	*P. vivax*
13	Hati [29]	Kolkata, India	Aug 2005-Dec 2010	Cross sectional	2971	46	1.5	NM	Blood smear /IgM and IgG ELISA	*P. vivax*-28, *P. falciparum*-18
14	Kaushik [30]	Dehradun, India	2006	Case report	1	1	NA	26	Blood smear/ IgM and IgG ELISA	*P. vivax* + *P. falciparum*
15	Malhotra [31]	Patiala, India	2012	Case report	1	1	NA	27	Blood smear /NS1 and IgM ELISA	*P. vivax*
16	Mittal [32]	Dehradun, India	Dec 2012- Nov 2013	Retrospective observational study	2547	8	0.3	Above 18	Blood film, RDT/IgM, NS1 ELISA	NM
17	Mohapatra [33]	Odisha, India	June-Sep 2011	Prospective observational study	469	27	6	NM	Blood smear /IgM and NS1 ELISA	*P. falciparum*-24, *P. vivax* – 2, *P. falciparum* + *P. vivax* - 1
18	Mørch [34]	Assam, Bihar, Chhattisgarh, Maharashtra, Anantpur Tamilnadu India	April 2011–November 2012	Cross sectional	1564	58	3.7	34 mean age	Blood smear/IgM, NS1 ELISA/	NM
19	Mushtaq [35]	Srinagar, infected in Delhi, India	Oct - 2012	Case report	1	1	NA	25	Blood smear, RDT/ IgM ELISA	*P. falciparum* + *P. vivax*
20	Pande [36]	Meerut, India	2013	Case report	1	1	NA	25	Blood smear /NS1 and IgM ELISA	*P. falciparum, P. vivax*
21	Raja [37]	Chennai, India	May 2013- Jan 2014	Cross sectional	100	3	3	NM	Blood smear/ELISA	NM
22	Rani [38]	Hyderabad, India	2015	Case report	1	1	NA	30s	Blood smear/IgM ELISA	NM
23	Rao [39]	Odisha (Angul), India	Jan-Dec 2013	Cross sectional	1980	22	1	All ages	Blood smear, RDT/ IgM and NS1 ELISA, PCR	*P. falciparum*- 12, *P. vivax-* 10
24	Singh [40]	Dehradun, India	July-Nov 2013	Retrospective	1141	9	0.8	12–80	Blood smear/IgM, NS1 ELISA	NM
25	Saksena [41]	Delhi, India	2017	Case report	1	1	NA	17 male	RMAT, PCR/IgM ELISA	*P. vivax, P. falciparum*
26	Singla [42]	Chandigarh, India	Jan 2011-Dec 2012	Cross sectional	300	1	0.3	NM	NM/NS1 and IgM ELISA	*P. vivax*
27	Shah [43]	Ahmedabad, India	June 2013-Nov 2014	Retrospective	8364	27	0.3	NM	Blood smear/NS1, IgM ELISA	*P. vivax* + DENV-17, *P. falciparum* + DENV-9, P. *falciparum* + P. *vivax* + DENV-1
28	Thangaratham [44]	Alappuzha, Kerala	2006	Case report	1	1	NM	22	Blood smear /IgM ELISA	*P. vivax,* DENV2
29	Yasir [45]	Karachi, Pakistan	April 2013-Jan 2014	Cross sectional	159	5	3	15–53 years	Blood smear /IgM ELISA	NM
Africa										
30	Ayorinde [46]	Ogun, Nigeria	April-May 2014	Cross sectional	60	1	2	All ages	Blood smear, RDT, PCR/NS1, IgM and IgG ELISA	*P. falciparum*
31	Baba [47]	Nigeria	July-Dec. 2008	Cross sectional	310	18	6	All ages	Blood smear /PRNT	*P. falciparum*
32	Charrel [48]	France, infected in Guinea, Senegal and Sierra Leone	2004, march	Case report	1	1	NA	37	Blood smear /IgM and IgG ELISA	*P. falciparum,* DENV3
33	Chipwaza [49]	Morogoro, Tanzania	March–May and Aug-Oct. 2013	Cross sectional	364	31	9	2–13	Blood smear /IgM and IgG ELISA, PCR	NM
34	Dariano [50]	Bo, Sierra Leone	2012–2013	Cross sectional	1260	3	0.2	All ages	RDTs/IgM, IgG, NS1 ELISA	NM
35	Kolawole [51]	Ilorin, Nigeria	2016	Cross sectional	176	5	3	All ages	RDT/IgM ELISA, PCR	DENV2, DENV3, DENV4
36	Oyeoro [52]	Ibadan, Nigeria	Jan-April 2013	Cross sectional	188	19	10	All ages	NM/IgG, IgM, NS1 ELISA	NM
37	Sow [53]	Kedougou, Senegal	July 2009–March 2013	Cross sectional	13,845	1	0.01	All ages	Blood smear, RDT/ IgM ELISA, PCR	*P. falciparum*
38	Stolar [54]	Ghana	2011–2014	Retrospective analysis	218	7	3	2–14 years	RDT/IgM and IgG, ELISA, PCR	*P. falciparum*
39	Vu [55]	Kenya	2016	Cross sectional	579	33	6	1–17 years	Blood smear /PCR	NM
Caribbean										
40	Serre [56]	Spain, Infected in Haiti	2011	Case report	1	1	NA	27	Blood smear, PCR/IgM, IgG and NS1 ELISA, PCR	*P. falciparum,* DENV4
Southeast Asia										
41	Che rahim [57]	Kelantan, Malaysia	2017	Case report	1	1	NA	59	Blood smear, PCR/NS1 ELISA	*P. knowlesi*
42	Chong [58]	Malaysia	2017	Case report	1	1	NA	59	Blood smear/NS1 and IgM ELISA	*P. knowlesi*
43	Issaranggoon [59]	Thailand	2014	Case report	1	1	NA	11	Blood smear/ NS1, IgM ELISA	*P. falciparum*
44	McGready [60]	Thai-Burmese border	Jan 2004-May 2006	Cross sectional	209	1	0.5	Pregnant women	Blood smear/IgM ELISA, NS1 ELISA	*P. falciparum, P. vivax*
45	Mueller [61]	(Oun Kouma, Ou Chra, Snoul) Rural Cambodia	Jan 2008- Dec 2010	Prospective observational study	1193	30	2.5	7–49 years	RDT/PCR	*P. falciparum, P. vivax*
46	Thaha [62]	Surabaya, Indonesia	Nov 2008	Case report	1	1	NA	NM	Blood smear/IgM, IgG ELISA	NM
47	Ward [63]	East Timor	2006	Case report	1	1	NA	7	Blood smear /IgM ELISA	*P. falciparum*
48	Yong [64]	Riau Island Indonesia	2012	Case report	1	1	NA	49	Blood smear/IgM, NS1 ELISA	*P. falciparum*
South America										
49	Carme [65]	French Guiana	July 2004-June 2005	Retrospective analysis	1723	17	1	NM	Blood smear/PCR, IgM ELISA, virus isolation	*P. vivax* − 14, *P. falciparum*- 3, DENV3–5, DENV1–1, NM-11
50	Epelboin [66]	French Guiana	2004–2010	Retrospective matched pair study	NM	104	NA	All ages	Blood smear/PCR, NS1, IgM, IgA ELISA	*P. vivax* – 80, *P. falciparum* – 21, *P. vivax* + *P. falciparum* – 3, DENV1–3, DENV2–2, DENV3–5, NM-94
51	Lupi [67]	Rio de Janeiro, Brazil	Apr 2013	Case report	1	1	NA	52	Blood smear, RDT, PCR/ IgM and NS1 ELISA, PCR	*P. ovale wallikeri*
52	Magalhaes [68]	Brazilian Amazon Manaus Brazil	March 2009 to April 2010	Retrospective study	132	11	8	Mean age, 42.7 yrs	Blood smear, PCR/NS1 ELISA, PCR	*P. vivax* DENV2, DENV3, DENV4
53	Magalhaes [69]	Brazilian Amazon Manaus Brazil	2009–2011	Cross-sectional	1578	44	3	All ages	Blood smear, PCR/ NS1 ELISA, PCR	*P. vivax*
54	Mendonca [70]	Brazilian Amazon Manaus Brazil	2009–2013	Prospective observational study	All febrile patients	30	NA	31.11 median age	Blood smear, PCR/ IgM and NS1 ELISA	*P. vivax,* DENV4–8, DENV3–1, DENV2–18, DENV1–3
55	Santana [71]	Novo Repartimento (Pará), Brazil	May 2003 to August 2005	Cross sectional	111	2	2	> 18 years	Blood smear/PCR	*P. vivax,* DENV2

N – sample size, ML/DN - Malaria/Dengue coinfection, ELISA - Enzyme linked immunosorbent assay, NS1 - Dengue non-structural protein − 1, PCR - Polymerase Chain reaction, RDT - rapid diagnostic test, PRNT - Plaque reduction neutralisation test, RMAT - Rapaid malaria antigen test, NM - not mentioned, NA - not applicable

**Table 2:** Coinfection cases of Dengue and Chikungunya

S.No.	Citations	Place	Year	Study design	N	Positive for coinfection	Coinfection (%)	Age	Diagnostic test DN/CK	Remarks
South Asia										
1.	Afreen [72]	Delhi, India	2014	Cross sectional	87	9	10	All ages	NS1, IgM, IgG ELISA, PCR/ IgM ELISA, PCR	DENV2 + CHIKV-5, DENV3 + CHIKV -2, DENV1 + CHIKV-1, DENV1 + DENV2+ CHIKV-1
2.	Carey [73]	Vellore, India	1964	Cross sectional	477	8	2	All ages	Virus isolation Serological Complement fixation and Hemagglutination inhibition assay for both infection	NM
3.	Chahar [74]	Delhi, India	2006	Cross sectional	69	6	9	All ages	PCR/PCR	DENV1, DENV3, DENV4
4.	Galate [75]	Mumbai, Maharashtra	April 2012-Oct. 2013	Cross sectional	200	19	10	13–60	IgM ELISA/IgM ELISA	NM
5.	Hapuarachchi [76]	Sri Lanka	2006	Case report	1	1	NA	70	PCR/PCR	NM
6.	Kalawat [77]	Tirupati, India	2011	Retrospective analysis	72	2	3	All ages	IgM ELISA / IgM ELISA	NM
7.	Kaur [78]	Delhi, India	Aug-Dec. 2016	Cross sectional	600	152	25	11–68	IgM ELISA, NS1 ELISA, PCR/IgM ELISA, PCR	NM
8.	Londhey [79]	Mumbai, India	June 2010–April 2015	Prospective observational study	300	30	10	All ages	IgM ELISA, PCR/ IgM ELISA, PCR	NM
9.	Mørch [34]	Assam, Bihar, Chhattisgarh, Maharashtra, Anantpur, Tamilnadu India	April 2011–November 2012	Cross sectional	1564	25	1.6	34 mean age	IgM, NS1 ELISA/IgM ELISA	NM
10.	Mukherjee [80]	Kolkata, India	July 2014-Oct. 2015	Cross sectional	326	53	16	All ages	IgM and NS1 ELISA, PCR/IgM ELISA, PCR	DENV2, DENV4
11.	Neeraja [81]	Hyderabad, Telangana	2007	Cross sectional	713	8	1	NM	IgG, IgM, PCR/PCR	NM
12.	Paulo [82]	Potugal, Infected in India	2016	Case report	1	1	NA	65	PCR/IgM ELISA	DENV3
13.	Rahim [83]	Dhaka, Bangladesh	2017	Case report	1	1	NA	23 female	NS1 ELISA/IgM ELISA	NM
14.	Saswat [84]	Khurda, Odisha Aurangabad, Maharashtra India	July-Dec. 2013	Cross sectional	222	43	19	All ages	NS1, IgM, IgG ELISA, PCR/IgM ELISA, PCR	DENV2
15.	Shaikh [85]	Karnataka, India	July 2010–June 2013	Cross sectional	6554	532	8	NM	IgM ELISA/IgM ELISA	NM
16.	Schilling [86]	Chennai, India	September 2008	Case report	1	1	NA	25	NS1, IgM ELISA and IFA/IgM IFA	NM
17.	Taraphdar [87]	West Bengal, India	2010	Cross sectional	550	68	12	All ages	IgM ELISA, PCR / IgM ELISA, PCR	DENV2, DENV3
18.	Kularatne [88]	Peradeniya, Srilanka	Dec. 2006-March 2007	Cross sectional	54	3	5	15–74	IgM ELISA, Hemagglutination inhibition/ IgM ELISA, Hemagglutination inhibition	NM
Africa										
19.	Baba [47]	Nigeria	July-Dec. 2008	Cross sectional	310	63	20	All ages	PRNT/PRNT	NM
20.	Caron [89]	Gabon	Sep 2007-Aug 2010	Cross sectional	4287	37	1	All ages	PCR of partial E gene/ PCR of partial E1 gene	DENV2
21.	Dariano [50]	Bo, Sierra Leone	2012–2013	Cross sectional	1260	13	1	All ages	IgM, IgG, NS1 ELISA/IgM ELISA	NM
22.	Leroy [90]	Gabon	March–July 2007	Cross sectional	773	8	1	NM	PCR/ PCR	DENV2
23.	Nkoghe [91]	Franceville, Gabon	Feb-July 2010	Cross sectional	433	20	4.6	1–77	PCR/PCR	NM
24.	Parreira [92]	Portugal, infected in Luanda, Angola	January 2014	Case report	1	1	NA	Early 50s	NS1 IgM, IgG ELISA, PCR/IgM ELISA, PCR	DENV4
25.	Ratsitorahina [93]	Tomasina, Madagascar	Jan-March 2006	Cross sectional	55	10	18	NM	IgM ELISA, PCR/IgM ELISA, PCR	DENV1
Caribbean										
26.	Edwards [94]	Guatemala	June 2015	Surveillance report	144	46	32	All ages	PCR/ PCR	DENV1–4, DENV2–40, DENV4–2
27.	Omarjee [95]	Island of Saint Martin	Dec. 2013- Jan 2014	Cross sectional	1502	16	1	All ages	IgM, IgG ELISA and PCR / IgM, IgG ELISA and PCR	DENV1–10, DENV2–2, DENV4–4
Southeast Asia										
28.	Cha [96]	Osong korea Infected (2 in Philllipine, 1 Vietnam, 1 Indonesia, 1 East Timor)	2009–2010	Cross sectional	486	5	1	11–70	IgM ELISA, PCR/ IgM ELISA, PCR	NM
29.	Chang [97]	Taipei China, infected in Singapore	2009 April	Case report	1	1	NA	12	IgM and IgG ELISA, PCR/ IgM and IgG ELISA, PCR	DENV2
30.	Khai Ming [98]	Rangoon, Burma	July 1970-Dec. 1972	Cross sectional	2060	55	2.6	0–11	HI, CF/HI, CF	NM
31.	Laoprasopwattana [99]	Southern Thailand	April–July 2009	Prospective Cohort study	50	1	2	≤15	IgM ELISA and Hemagglutination inhibition/IgM IFA, PCR	NM
32.	Nayar [100]	Kinta, Malaysia	2006	Case report	2	2	NA	22 and 28	NS1, IgM ELISA, PCR/PCR	DENV1
33.	Ooi [101]	Selangor, Malaysia,	2009	Case report	1	1	NA	NM	NM/Complete Genome sequencing of CHIKV	DENV2
34.	Phommanivong [102]	Champasak Laos	July-Aug 2013	Cross sectional	40	5	12.5	5–65	PCR/PCR	DENV2–3, DENV3–2
35.	Tun [103]	Mandalay, Myanmar	July–October 2010	Cross sectional	116	7	6	≤12	IgM ELISA, PCR/IgM ELISA, PCR	NM
North America										
36.	Kariyawasam [104]	Toronto, Canada	May 2006-April 2007 and Feb 2013-March 2014	Retrospective analysis	1304	1	0.07	0–91	PCR/PCR	DENV-1
37.	Lindholm [105]	Maryland, USA	Dec 2013-May 2015	Cross sectional	267	2	0.7	25–60	IgM, IgG ELISA, PCR, PRNT/ IgM, IgG ELISA, PCR, PRNT	NM
South America										
38.	Bocanegra [106]	Barcelona Spain Infected in south America	April 2014–2015	Retrospective	42	5	12	34.6 mean age	IgM ELISA/IgM ELISA, PCR	NM
39.	Brooks [107]	Santos, Brazil	2017	Case report	1	1	NA	27	IgM ELISA/IgM ELISA	NM
40.	Calvo [108]	Girardot, Colombia	Feb 2015	Cross sectional	8	4	50	0–10	IgM ELISA, PCR/PCR	NM
41.	Carrillo-Hernández [109]	Norte de Santander, Colombia	August 2015 – April 2016	Cross sectional	157	12	7.6	26.81	PCR/PCR	NM
42.	Farrell [110]	Machala, Ecuador	2015	Case report	1	1	NA	35	IgM, IgG ELISA/PCR	NM
43.	Gomez-Govea [111]	Nuevo leon, Mexico	Jan-Oct 2015	Cross sectional	101	5	5	31 median age	IgM ELISA/IgM ELISA, PCR	NM
44.	Mercado [112]	Bogota, Colombia	Sept 2014-Oct 2015	Retrospective analysis	58	7	12	NM	IgM ELISA, PCR/PCR	NM
45.	Rosso [113]	Cali, Colombia	2015	Case report	1	1	NA	72	PCR/ PCR	DENV3
Middle East										
46.	Malik [114]	Al-Hudaydah, Yemen	Oct 2010-March 2011	Cross sectional	136	1	0.7	NM	IgM ELISA, PCR/IgM ELISA	NM
47.	Rezza [115]	Al-Hudaydah Yemen	2012	Cross sectional	400	14	3.5	All ages	IgM, IgG ELISA and PCR/ IgM, IgG ELISA and PCR	DENV2 Predominantly

N – sample size, DN/CK – Dengue/Chikungunya coinfection, ELISA – Enzyme linked immunosorbent assay, NS1 - Dengue non-structural protein −1, PCR – Polymerase Chain reaction, IFA – immunofluorescence assay, PRNT – Plaque reduction neutralisation test, NM – not mentioned, NA – not applicable

**Table 3:** Coinfection cases of Malaria and Chikungunya

S.No.	Citations	Place	Year	Study design	N	Positive for coinfection	Coinfection(%)	Age	Diagnostic test ML/CK	Remarks
South Asia										
1.	Mørch [34]	Assam, Bihar, Chhattisgarh, Maharashtra, Anantpur, Tamilnadu	April 2011–Nov 2012	Cross sectional	1564	20	**1.3**	34 mean age	IgM, NS1 ELISA/IgM ELISA	NM
Africa										
2.	Ayorinde [46]	Ogun, Nigeria	April-May 2014	Cross sectional	60	9	15	All ages	Blood smear, RDT, PCR/IgM ELISA	*P. falciparum*
3.	Baba [47]	Nigeria	July-Dec. 2008	Cross sectional	310	21	6.7	All ages	Blood smear /PRNT	*P. falciparum*
4.	Chipwaza [49]	Morogoro, Tanzania	March–May and Aug-Oct. 2013	Cross sectional	364	2	0.6	2–13 years	Blood smear / IgM and IgG ELISA,	NM
5.	Dariano [50]	Bo, Sierra Leone	2012–2013	Cross sectional	1260	118	9	All ages	RDTs/IgM ELISA	NM
6.	Mugabe [116]	Quelimane Mozambique	Feb-June 2016	Cross Sectional	163	2	1.2	28 median age	RDT /IgM ELISA, PCR	NM
7.	Sow [53]	Kedougou, Senegal	July 2009–March 2013	Cross sectional	13,845	3	0.02	All ages	Blood smear, RDT/ IgM ELISA, PCR	*P. falciparum*

N – sample size, ML/CK- Malaria/Chikungunya coinfection, ELISA – Enzyme linked immunosorbent assay, NS1 - Dengue non-structural protein −1, PCR – Polymerase Chain reaction, RDT – rapid diagnostic test, PRNT – Plaque reduction neutralisation test, NM – not mentioned

**Table 4:** Coinfection cases of Malaria, Dengue and Chikungunya

S.No.	Citations	Place	Year	Study design	N	Positive for coinfection	Coinfection (%)	Age	Diagnostic test ML/DN/CK	Remarks
South Asia										
1.	Abdullah [117]	Delhi, India	2016	Case report	1	1	NA	21	Blood smear, RDT/PCR/IgM ELISA, PCR	*P. vivax,* DENV3
2.	Gupta [118]	Delhi, India	2017	Case report	1	1	NA	55	RDT/NS1, IgM ELISA/PCR	*P. falciparum*
3.	Mørch [34]	Assam, Bihar, Chhattisgarh, Maharashtra, Anantpur, Tamilnadu India	April 2011–Nove 2012	Cross sectional	1564	2	0.1	34 mean age	Blood smear/IgM, NS1 ELISA/IgM ELISA	NM
4.	Tazeen [119]	Delhi, India	2016	Case report	1	1	NA	3	Blood smear /PCR/PCR	*P. vivax*
Africa										
5.	Dariano [50]	Bo, Sierra Leone	2012–2013	Cross sectional	1260	4	0.3	All ages	RDTs/IgM, IgG, NS1 ELISA/IgM ELISA	NM
6.	Raut [120]	India Infected in Nigeria	2014	Case report	1	1	NA	21	Blood smear / NS1 ELISA, PCR/PCR	*P. falciparum*

N – sample size, ML/DN/CK – Malaria/Dengue/Chikungunya coinfection, ELISA – Enzyme linked immunosorbent assay, NS1 - Dengue non-structural protein −1, PCR – Polymerase Chain reaction, RDT – rapid diagnostic test, NA – not applicable, NM-not mentioned

Out of the 55 reports about Malaria/Dengue coinfections, only ten have reported the serotype of the Dengue virus. Out of the 47 reports about Dengue/Chikungunya coinfections 20 reports have mentioned the serotype of Dengue virus. Earliest report of Malaria/Dengue coinfection came in 2003 from Brazil, while earliest reported case of Dengue/Chikungunya coinfection came in 1964 from India. Malaria/Chikungunya cases were reported as late as 2008 from Nigeria. A retrospective matched pair study from French Guiana reported most cases (104) of Malaria/Dengue coinfections. Maximum cases of Dengue/Chikungunya coinfections (532) were reported from Karnataka in India and most cases of Malaria/Chikungunya coinfections (118) were reported from Bo, Sierra Leone.

**Fig. 2:**
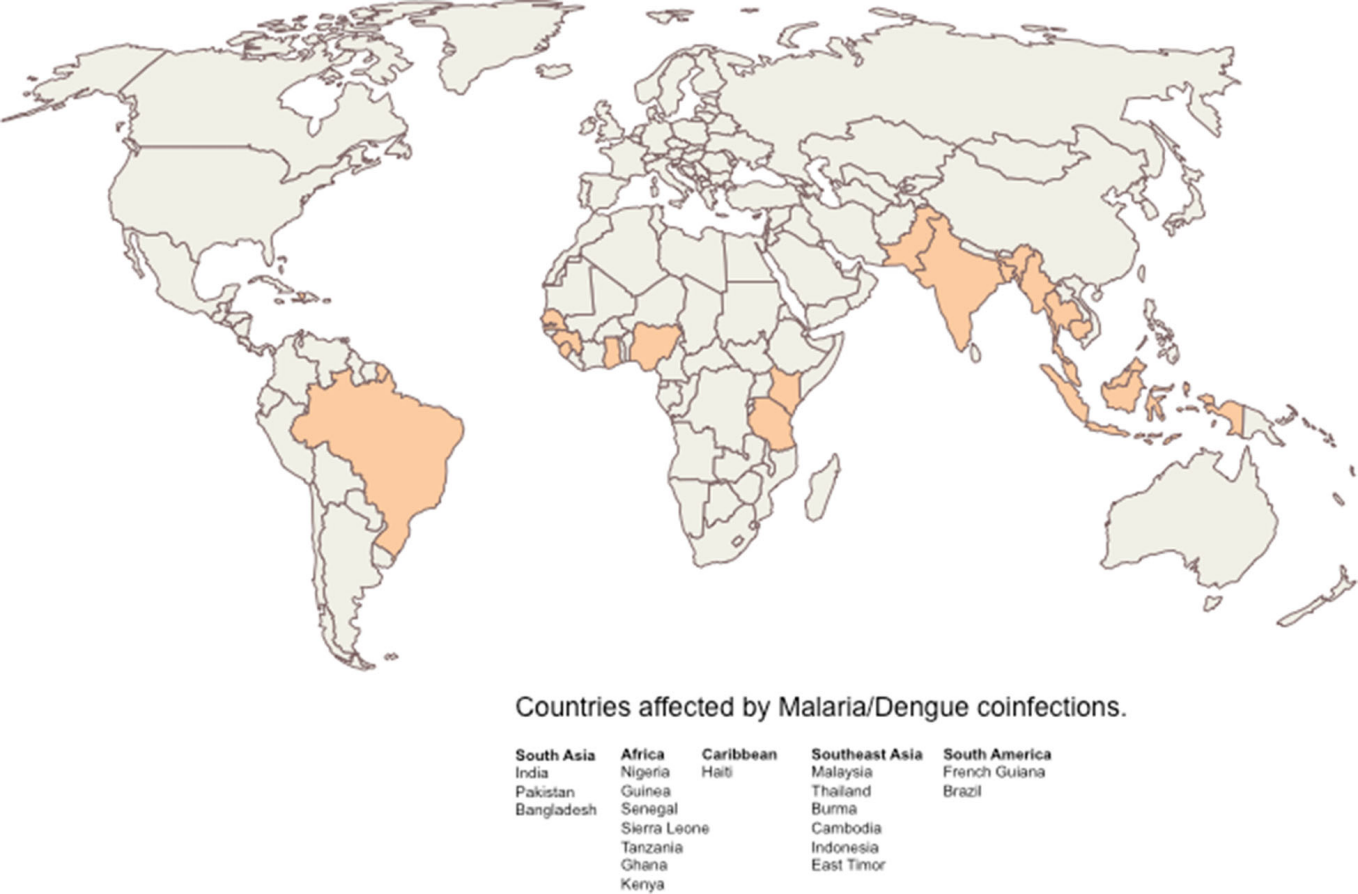
Global distribution of Malaria/Dengue coinfections

**Fig. 3:**
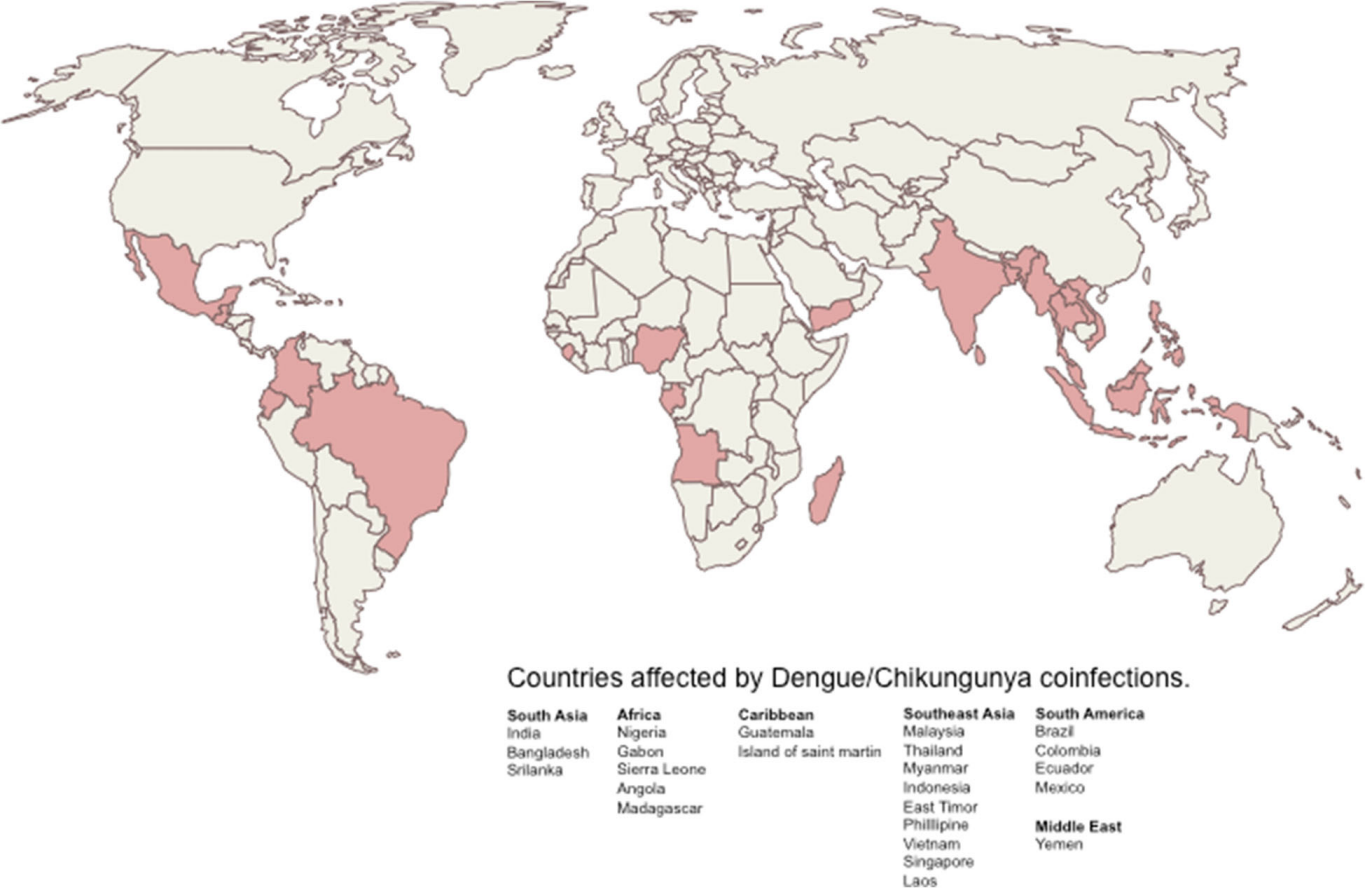
Global distribution of Dengue/Chikungunya coinfections

**Fig. 4:**
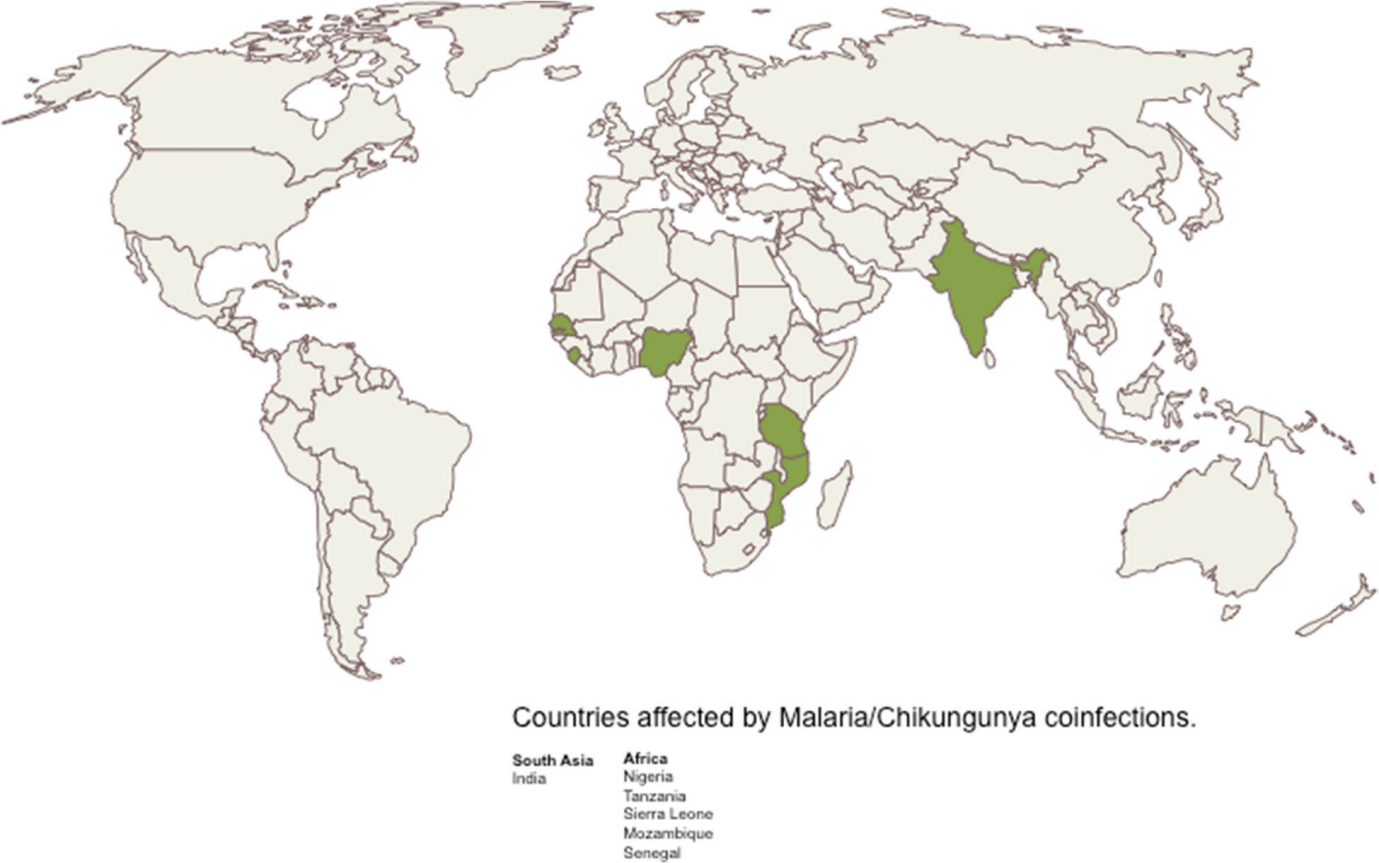
Global distribution of Malaria/Chikungunya coinfections

**Fig. 5:**
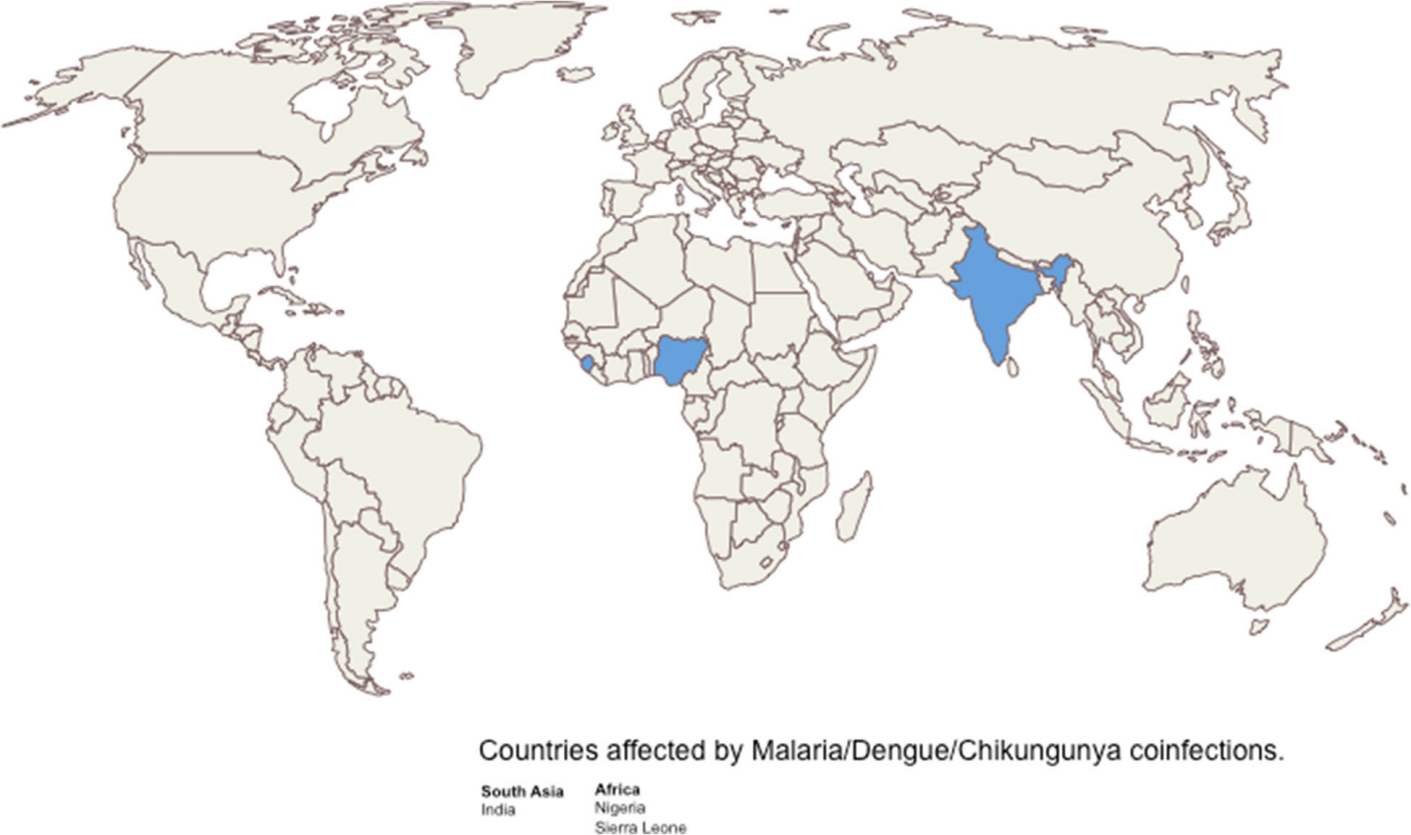
Global distribution of Malaria/Dengue/Chikungunya coinfections

**Fig. 6:**
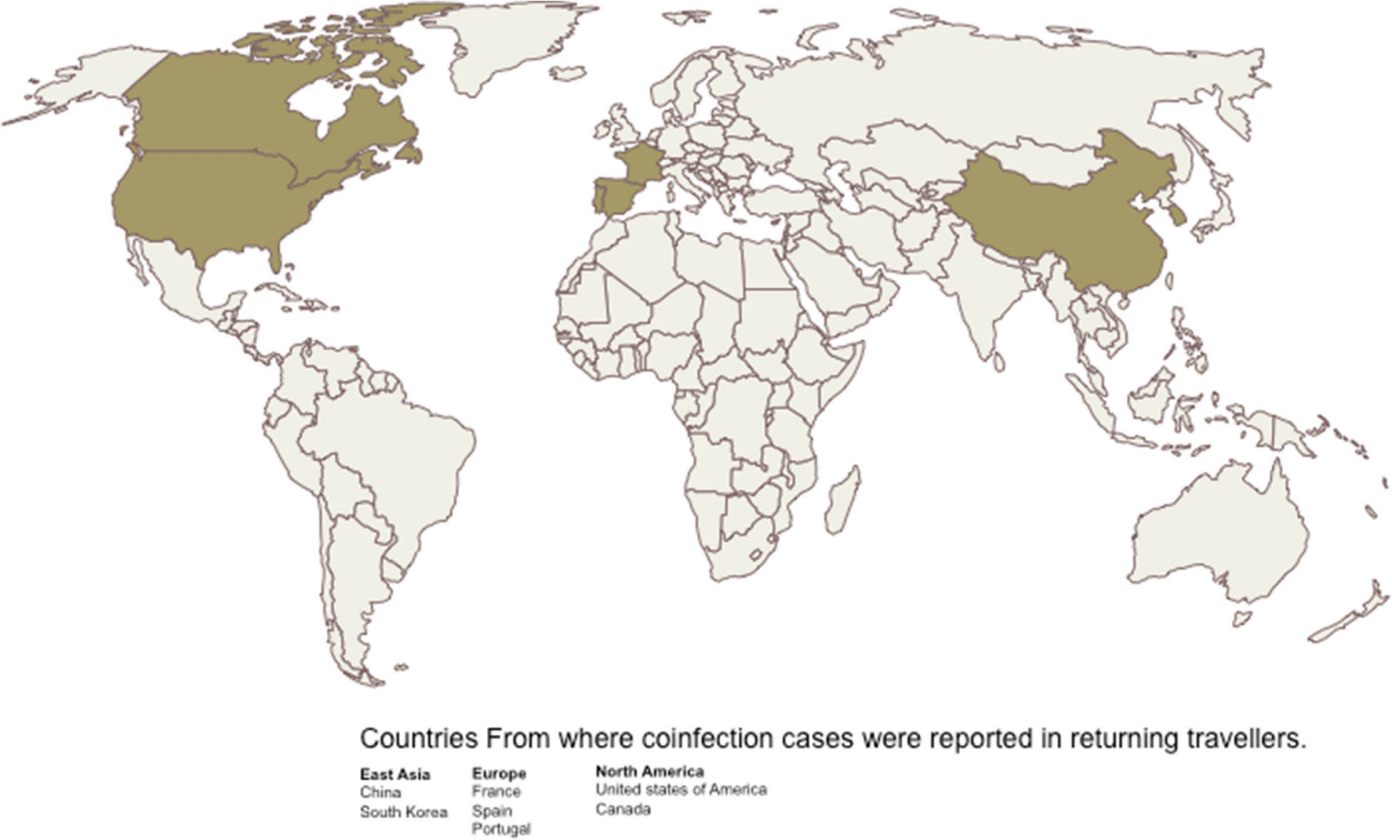
Countries from where coinfection cases were reported in returning travellers

Most cases of coinfections were reported from South Asia (52), primarily from India, followed by Africa (25), South-east Asia (16), South America (15), Caribbean (3) and Middle East (2). Two studies from North America reported coinfections of Dengue/Chikungunya in returning travellers without identifying the location where coinfections occurred. Malaria/Dengue coinfections were reported from 44 unique locations spread across 20 different countries (Fig. 2). Dengue/Chikungunya coinfections were reported from 48 unique locations spread across 26 countries (Fig. 3). 5 countries from African continent and India reported cases of Malaria/Chikungunya coinfections (Fig. 4). Cases of Malaria/Dengue/Chikungunya coinfections were reported from India, Sierra Leone and Nigeria (Fig. 5). Seven countries reported infection in returning travellers(Fig. 6). Based upon cross sectional studies Malaria/ Dengue prevalence varied widely, ranging between 0.1–23% from south Asia, 0.01–9% from Africa, 0.5–2.5% from Southeast Asia and 1–3% from South America. The frequency of Dengue/Chikungunya coinfections ranged from 1 to 25% from South Asia, 1–20% from Africa, 1–32% from Caribbean, 1–12.5% from Southeast Asia, 0.07–0.7% from North America, 5–50% from South America and 0.7–3.5% from Middle east. Malaria/Chikungunya coinfections frequency ranged from 0.02–15% from Africa and a single study reported from India reported 1.3% patients coinfected with both pathogens. Malaria/Dengue/Chikungunya coinfection frequency was reported by two cross sectional studies, one from India with 0.1% prevalence and another from Sierra Leone with 0.3% prevalence.

## Discussion

Malaria, Dengue and Chikungunya are arthropod borne diseases that have shared endemic profiles. These diseases are spread by mosquito vector, which are found in abundance in tropical regions of the world. *Anopheles* mosquito, which transmits Malaria parasite, is a night biting mosquito and breed in stagnant water [121]. *Aedes* that spreads Dengue and Chikungunya, on the other hand bites in daylight and breeds in stored clean water [122]. Expansion of the *Aedes* vector has lead to introduction of Dengue and Chikungunya to newer locations. Rapid urbanisation without the development of civic infrastructure, constant movement of population for livelihood, monsoon dependent breeding patterns and overlapping habitats have lead to co-circulation and coinfection of these pathogens in the same population [123]. Diagnosis of cases of coinfection is compounded by the fact that initial symptoms of all three diseases are very similar that include febrility as the common factor. Several reports have been published that does not identify the coinfecting pathogen due to lack of distinguishing symptoms at the time, but retrospective analysis later revealed otherwise. In resource poor settings and during outbreaks clinicians might not have the resources or time to rely on detailed investigations.

We have attempted to identify regions of the world from where cases of mixed infection with Malaria, Dengue and Chikungunya have been reported. We searched the databases to identify published reports about any of these coinfections. Most reports of Malaria/Dengue and Dengue/Chikungunya coinfections were reported from India. In recent years there have been many outbreaks of Dengue and Chikungunya in India, not to mention that the first published report of Dengue/Chikungunya coinfection was reported from India in 1967 [72]. However the overall percentage of Malaria/Dengue coinfections was low which, can be explained by different vector species for Malaria verses Dengue and Chikungunya. The highest frequency of Malaria/Dengue coinfections was reported from Pakistan that is endemic for both Malaria and Dengue. Lowest frequency was reported form Senegal with only 0.01%. 41 reports clearly identified the parasite species for Malaria infection but only 10 reported the serotype of Dengue virus. All four serotypes were found to exist with Malaria parasite. Coinfection cases were found in all age groups and gender. Nearly 85% of the reports for Malaria/Dengue coinfections have used microscopic confirmation of the Malaria parasite identifying the parasite load and species. Dengue infections were primarily detected by a combination of immunoglobulin ELISA, NS1 ELISA and PCR.

Dengue/Chikungunya coinfections were reported by 47 studies and an overall higher percentage as compared to Malaria/Dengue coinfection possibly because of similar vector species. The Highest frequency of Dengue/Chikungunya coinfections was reported from Colombia and lowest from Canada in returning travellers. Dengue virus serotype-4 was the predominant serotype found in cases of coinfections. Malaria/Chikungunya coinfections were rare with only 7 published reports. All of them were reported from Africa and India. 6 studies reported Malaria/Dengue/Chikungunya coinfections, four of them were case reports and two cross sectional studies. Three of the case reports were infected in Delhi while another one could have been infected in Nigeria or India. Delhi has become a hub of Industrial and social activities with a burgeoning population. Almost every year during monsoon season the city witnesses Dengue outbreaks with thousands of people getting infected. Due to the lack of distinguishing clinical features, laboratory diagnosis based on endemic patterns and outbreak reports are the only way for adequate clinical management of double or triple coinfections. At least 12 studies reported coinfections in returning travellers underlining the role of travel-based spread of the diseases. This phenomenon has been observed for SARS, MERS-CoV and Dengue [124–126]. Exposing a naïve population to new pathogens might lead to disease outbreak, not to mention viral mutations to adapt its human or mosquito host resulting in more pathogenic strain. Travel advisories and routine surveillance of returning travelers to endemic regions should be implemented stringently to control spread of infections.

Interaction of multiple pathogens within a host may potentially result in several different outcomes. Firstly, if the coinfecting organisms are dependent on similar tissues, the host may have to deal with multiple pathogens at the same time and place. Such interactions are likely to be detrimental to the host as happens in the case of coinfection with Hepatitis B, C and Delta virus coinfections. Hepatitis B, C and Delta virus coinfections results in severe chronic disease that responds poorely to the interferon alpha treatment [127] as compared to single infections. Secondly, the immune effector mechanisms triggered by one pathogen may weaken or divert the host immunity leading to severe outcomes or increased resistance to therapy as exemplified in the case of infection with *Mycobacterium tuberculosis* and parasite coinfections [128]. Thirdly, the coinfection may not have any serious effect on the prognosis of disease. However, even in such cases the misdiagnosis and mistreatment that may result, can be detrimental to the host. And finally, a coinfection may infact lead to better prognosis. For instance, it has been observed in the decreased mortality rate among the HIV patients coinfected with hepatitis G virus as compared to patients infected with HIV [129]. *Plasmodium*, Dengue virus and Chikungunya virus all infect different cell types in humans and might influence immune effector mechanism by downregulationg proinflammaotry cytokines like IL-12 and IFN-γ [11, 130]. A proper clinical analysis of Malaria, Dengue and Chikungunya coinfection is necessary to form an informed opinion on following a treatment regimen that best supports the patient and leads to an early resolution of the infection. Out of 104 reports, there are very few reports that have actually looked at the disease severity by establishing proper controls and comparing it with cases of monoinfections systematically. For Malaria/Dengue coinfections, prolonged fever, thrombocytopenia, anemia, renal failure and Jaundice were more pronounced as compared to monoinfections. Dengue/Chikungunya coinfections can result in diarrahea, deep bleeding, hepatomegaly and overall increase in disease severity. High grade fever was the only distinguishing feature of Malaria/Chikungunya coinfection. More such studies are required to create a consensus about disease outcome in cases of coinfections. Animal models that can replicate the coinfection scenario would be very helpful in identifying severity patterns for these diseases.

The distribution of *Aedes* vector has been reported from Southeast Asia, South Asia, East, Central and West Africa, Caribbean and South America. *Aedes aegypti* and *Aedes albopictus* are responsible for the spread of Dengue, Chikungunya, West Nile, Yellow fever and Zika virus [131]. It is difficult to distinguish whether cases of coinfection are due to separate mosquito bites delivering the viruses or single bite by mosquito harboring both viruses. The incubation period of both viruses is nearly same so both diseases are manifested around the same time. *Anopheles* has also been reported from the above-mentioned regions and also from East and central Asia, Europe and North America [132]. Most cases of Malaria/Dengue and Malaria/Chikungunya coinfections were found from the regions where both vector species are present. In many instances a seasonal pattern of infections is observed with most cases being reported during monsoon season, which coincides with the breeding season of Mosquito vector. Climatic, sociodemographic and environmental factor play a crucial role in survivability and distribution of the mosquito vector thereby influencing cases of coinfections [133]. Vector control continues to be an integral part of reducing disease burden but very few studies reported about the vectors responsible for cases of coinfection. Routine collection of vector surveillance data and thorough analysis of the role of vectors in coinfection cases need to be assessed.

Data collection is prone to bias, to this affect we have made every effort to search and analyze the current literature with broad search queries, nonetheless many relevant studies were unavailable due to lack of full text availability. Also the review relied completely on published literature where grey literature and studies with minimal or negative results may not have been included resulting in publication bias. Furthermore, studies obtained were of variable quality and many did not reported data on disease severity and outcomes in cases of coinfections. Despite these lacunas, the present study attempts to clearly identify regions of the world from where cases of coinfections were reported by thorough search and analysis of published reports. Our analysis indicates that coinfections with Malaria, Dengue and Chikungunya or in rare instances all three is a possibility. Our analysis also indicates that there are higher percentages of people with febrile symptoms, which might have Dengue/Chikungunya coinfections as compared to Malaria/Dengue or Malaria/Chikungunya coinfections. Shared epidemiology, vector distribution and co-circulation of pathogens are some of the reasons for coinfections. We have georeferenced cases of coinfections and identified affected countries of the worlds, establishing co-endemicity of these infections, which might help in proper and complete diagnosis of cases of coinfections with similar initial symptoms.

## Conclusion

This systematic review has found evidence of Malaria, Dengue and Chikungunya coinfections in 42 Countries spread across several geographical locations. Malaria/Dengue was the most prevalent coinfection followed by Dengue/Chikungunya. These infections often affect same populations due to share endemicity and can be present simultaneously in the same individual. Similar initial symptoms make it harder for clinicians to identify cases of coinfections. Most coinfections were found from South Asia and Africa. *P. falciparum* and *P. vivax* were the most common malaria species found with coinfecting arbovirus and DENV-4 was the most common serotype found in cases of Dengue coinfections. Prolonged and high grade fever, thrombocytopenia, diarrhea, Jaundice and hepatomegaly were some of the signs and symptoms associated with cases of coinfection. We also found evidence of coinfections in returning travellers, which have the potential to introduce the pathogen into new locations with established vector populations. Our study highlights the global prevalence of cases of coinfection and their geographical distribution, which could help in systematic planning, surveillance, diagnosis and health care delivery to the affected population.

## Additional file

Additional file 1: **Table S1.** Detailed search strategy. (DOCX 14 kb)

## Abbreviations

CHIKV: Chikungunya Virus
DENV: Dengue Virus
ELISA: Enzyme linked immunosorbent assay
MERS-CoV: Middle East respiratory syndrome corona virus
PCR: Polymerase chain reaction
SARS: Severe Acute Respiratory Syndrome
