# Supplementary material for: Global prevalence and distribution of coinfection of malaria, dengue and chikungunya: a systematic review
Source: BMC Public Health. 2018 Jun 8;18:710. doi: 10.1186/s12889-018-5626-z (PMC5992662; doi:10.1186/s12889-018-5626-z)
Supplement: Supplementary file 1 — Table S1. Detailed search strategy. (DOCX 14 kb) [file 12889_2018_5626_MOESM1_ESM.docx]

**Additional file 1: Table S1** Detailed Search Strategy

| **Database** | **Keywords** | **Results** | **Total** | **Unique reports (After removal of duplicates)** |
| --- | --- | --- | --- | --- |
| PubMed | Malaria AND Dengue | 1444 | 3355 | 2886 |
|  | Dengue AND Chikungunya | 1645 |  |  |
|  | Malaria AND Chikungunya | 266 |  |  |
| Web of Knowledge | Malaria AND Dengue | 1659 | 3965 | 3427 |
|  | Dengue AND Chikungunya | 1992 |  |  |
|  | Malaria AND Chikungunya | 314 |  |  |
